# Supplementary material for: Light Therapy for Cancer-Related Fatigue in (Non-)Hodgkin Lymphoma Survivors: Results of a Randomized Controlled Trial
Source: Cancers (Basel). 2021 Sep 30;13(19):4948. doi: 10.3390/cancers13194948 (PMC8508131; doi:10.3390/cancers13194948)
Supplement: Supplementary file 1 [file cancers-13-04948-s001.zip › cancers-1379326-supplementary.pdf]

Supplementary material 1 Specifics of light therapy devices

The Litebook Edge (Litebook, Ltc. Medicine Hat, Canada) is a small (15 x 12 x 1 cm) lightweight box that contains 60 premium white light emitting diode (LED) lights that mimic the visible spectrum of sunlight. Participants placed this device on a table at a distance of 45 cm in an angle of 45° from the face. The Luminette glasses contain 8 LEDs that are directed to the lens via a holographic field. Table S1.1 shows estimations of irradiance for both devices. Figure S1.1 and S1.2 show the light spectrum of both devices.

Table S1.1 Estimations of irradiance for the Litebook Edge and Luminette glasses.

	LB BWL lat	LB BWL cl	LB DWL lat	LB DWL cl	LUM BWL	LUM DWL
Peak spectral irradiance, nm	450	450	455	455	465	470
Visibility, lux	351.44	148.71	2.49	0.65	1012.75	8.37
S cone sensitivity, α-opic lux	530.57	228.54	3.36	0.9	1966.49	17.51
Melanopsin sensitivity, α-opic lux	373.99	160.55	2.59	0.69	1934.17	20.84
Rod sensitivity, α-opic lux	364.89	156.19	2.54	0.68	1639.64	16.84
M cone sensitivity, α-opic lux	357.48	152.01	2.52	0.67	1277.56	11.99
L cone sensitivity, α-opic lux	343.95	145.53	2.44	0.64	1056.78	9.2
Irradiance, $\mu W/cm^2$	126.05	53.6	0.9	0.26	438.74	4.1
Photon flux, $1/cm^2/s$	3.38E+14	1.43E+14	2.45E+12	7.45E+11	1.14E+15	1.06E+13
Log photon flux, log10 ($1/cm^2/s$)	14.53	14.16	12.39	11.87	15.06	13.02

Derived from: Lucas RJ, Peirson SN, Berson D, Brown T, Cooper H, Czeisler CA, Figueiro MG, Gamlin PD, Lockley SW, O'Hagan JB, Price LLA, Provencio I, Skene DJ, Brainard G. Irradiance Toolbox, 2013

Note: estimations of irradiance are based on the average values of five measurements performed with a radio spectrometer.

BWL = Bright White light; cl = contralateral eye (eye furthest from light source); DWL = Dim White Light; lat = lateral eye (eye closest to light source) LB = Litebook Edge; LUM = Luminette.

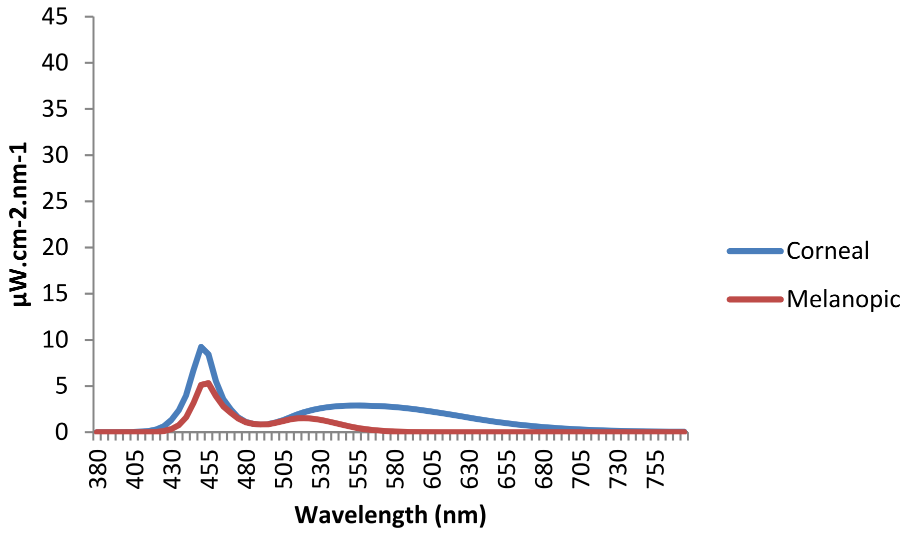

Figure S1.1. Light spectrum of the BWL Litebook Edge.

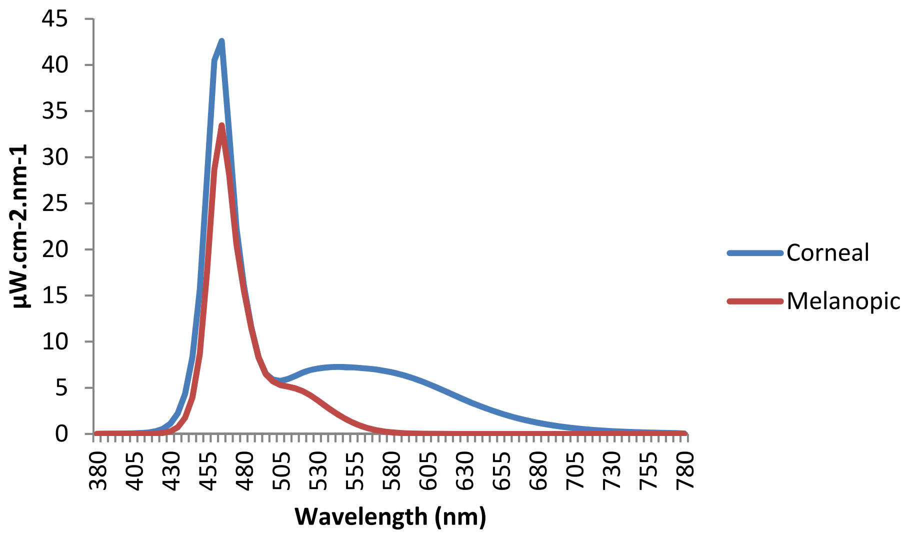

Figure S1.2. Light spectrum of the BWL Luminette glasses

Supplementary material 2: collection, handling, and processing of saliva samples

Methods

Procedure

All participants were asked to collect saliva on the Friday before the start of light therapy and the Friday after the end of light therapy to assess cortisol. Five saliva samples were collected at waking time, 30 minutes and 45 minutes after awakening, at 4 pm, and at bedtime. A subsample of 60 participants were asked to collect five additional samples at -5h, -4h, -3h, -2h, and -1h before bedtime to explore the effect of light therapy on melatonin secretion. Saliva was collected by a passive drool technique in a propylene vial. Participants were asked to avoid smoking, vigorous exercise, caffeinated and alcoholic drinks, chocolate, bananas, and food containing food colouring. Eating and drinking of other nourishments and brushing teeth was not allowed 15 and 30 min prior to sampling, respectively. Participants were instructed to note the time of sampling on a tracking sheet and store the samples in their home refrigerator. Samples were sent to the Netherlands Cancer Institute on the Monday after saliva collection. Samples were subsequently stored in a freezer at a -80°C until processing. Cortisol and melatonin values were determined using liquid chromatography - mass spectrometry (LC-MS). Cortisol and melatonin concentrations were calculated in nmol/l and pmol/l with a lower limit of quantitation of 0.5 nmol/l and 10.0 pmol/l, respectively.

Salivary cortisol and melatonin assay

Reagents were Melatonin d4; N-Acetyl-5-methoxytryptamine-α,α,β,β-d_4 from CDN Isotopes (Quebec, Canada), Cortisol C13; Cortisol-2,3,4- $^{13}C_3$, melatonin standard >98%, cortisone standard >98% and cortisol standard >98% from Sigma Aldrich (St. Louis, MO, USA).

Samples were prepared by adding a 250 µL salivary aliquot to a 2 mL Safe-lock Eppendorf tube and 10 µL of internal standard solution was added. Analytes were extracted by adding 1 mL of ethyl acetate, 15 minutes shaking of the samples, centrifuging the samples (5 minutes, RT, 8000g), snap freezing the hydrophilic phase and collecting and evaporating the organic phase. Next, the extracts were dissolved in 100 µL of injection fluid (20% methanol, 80% water) and spun down (5 minutes, RT, 12000g), before analysis. All samples were analyzed.

The cortisol and melatonin concentrations were analyzed in a single analytical measurement using liquid chromatography isotope-dilution tandem mass spectrometry. The Shimadzu Nexera X2 ultra high performance liquid chromatographer (Columbia, MD, USA) was employed to provide a flow of 0.6 mL/min through a C-18 Column (2,6 µm 50 mm x 2,10 mm) from Phenomenex (Torrance, California, USA). Column temperature was maintained at 30 °C. Chromatography was performed using a linear gradient between an aqueous phase containing 0.1% formic acid and 2 mM ammonium acetate and methanol. Here, a mobile phase of 30% methanol increased to 46.5% in 2.8 minutes, then mobile phase is changed to

100% methanol for 1.4 minutes before equilibrating tot the starting mobile phase containing 30% methanol.

Cortisol and melatonin quantitation was performed using multiple reaction monitoring mode on a QTRAP6500+ mass spectrometer (Sciex, Concord, ON, Canada). Ionization was achieved with an IonDrive™ Turbo V Source operated in positive mode. The mass transitions used for the analytes and their international standards (IS) are presented in Table S2.1.

Table S2.1. Applied MS/MS settings.

	Q1 massa (Da)	Q3 massa (Da)	Analyte
1	233,190	174,000	Melatonine Quan
2	233,190	159,000	Melatonine Qual
3	237,212	178,100	Melatonine d4 quan
4	237,212	163,200	Melatonine d4 qual
5	363,065	327,100	Cortisol quan
6	363,065	121,000	Cortisol qual
7	366,243	124,100	Cortisol C13 Quan
8	366,243	126,100	Cortisol C13 qual

Salivary cortisol and melatonin assay performance characteristics

The method was calibrated by weighted stock standards. A method comparison study with the published method from UMC Groningen (n = 20) confirmed proper method calibration (Table S2.2). Method imprecision was determined by running three control levels in quadruple in seven individual runs. The obtained imprecisions are presented in Tables S2.3.

Lower limit of quantitation (LLOQ) was based on lowest concentration with a CV of ≤ 20% and a Singal/Noise ratio of ≥ 10. The LLOQs were 0.010 nmol/L for melatonin and 0.50 nmol/L for cortisol. Samples with identifiable peaks below the LLOQ were quantified, but are associated with higher inaccuracy.

No interference was observed for DHEA, prednisone, androstenedione, 17-hydroxyprogesterone, progesterone, testosterone, 17β-estradiol, serotonin, L-tryoptophan, 5-OH-trytophan, aldosterone, 5-HIAA and N-actetyl-5-hydroxytryptamine. Prednisolone did interfere with cortisol, but this interference could be identified from the chromatogram and was not observed in the study samples.

Table S2.2. Method relation and correlation with other method [78].

	Cortisol	Melatonin
Slope	1.12	1.01
Intercept	0.398	0.0026
Pearsons' R^2	0.99	0.99

Table S2.3. Method imprecision for cortisol and melatonin.

	Average (nmol/L)	SD	CV (%)
Cortisol			
QC-high	39,3	5,46	13,9%
QC-intermediate	29,2	3,37	11,6%
QC-low	4,8	0,52	10,9%
Melatonin			
QC-high:	0,900	0,075	8,3%
QC-intermediate:	0,355	0,034	9,6%
QC-low:	0,049	0,006	11,6%

Data reduction

The five cortisol samples were used to determine the cortisol awakening response (CAR), diurnal slope, and total cortisol output. The *CAR* is the rapid increase in cortisol concentrations during the first 30 to 45 minutes after awakening [45]. The CAR mean increase (MnInc) was calculated using the formula: (awakening + 30 min + awakening + 45 min)/2 – awakening [79]. The *diurnal slope* reflects circadian fluctuations in cortisol [80]. It was determined by regressing the natural log-transformed cortisol values of awakening, 4 pm, and bedtime on time since waking (in hours). The unstandardized coefficient was used as a measure for diurnal slope. A flatter decline in cortisol levels over the course of the day is represented by larger (smaller magnitude negative) unstandardized values, while a steeper decline in cortisol values is represented by smaller unstandardized values. The *total cortisol output* reflects the exposure during the day [80] and was calculated as the area under the curve with respect to the ground (AUC_g) based on the trapezoidal formula using all five cortisol samples [81].

For the subsample that collected five additional samples at -5h, -4h, -3h, -2h, and -1h before bedtime, these 5 samples and the bedtime sample were used to determine the *dim light melatonin onset* (DLMO), which represents the start of the secretion of melatonin in dim light situations. A hockey-stick method was used to determine the DLMO using the hockey-stick program module designed [47]. For this purpose, melatonin concentrations were converted from pmol/l to pg/ml by dividing detected concentrations by 4.3.

Results

Participants

From the 166 participants, 155 participants agreed to collect saliva for the cortisol assessment. Seventeen participants were excluded from the cortisol analyses because of corticosteroid use leaving to a total sample of 138 participants. The availability of cortisol profiles at T0 (100%) and T1 (96%) were similar between groups. For an overview, see Consort diagram. A total number of 1334 samples were available for analysis. Thirty-six samples (2.7%) had undetectable cortisol levels and were imputed with the lower limit of quantitation divided by two (0.25 nmol/l). After screening for outliers (≥ 3.5 standard deviation), 23 (1.7%) samples were removed. Figure S2.1 shows the raw cortisol values at each sampling point per group at pre- and post intervention. As the CAR is easily underestimated when collection of the first samples is delayed by more than 15 minutes after awakening [82], the time point of the first collection was compared to actigraphy-derived awakening time. Therefore, 26 and 22 profiles were excluded at baseline and post-intervention, respectively due to a delay of ≤ 15 min between waking and first sample collection.

From the 155 participants that agreed to collect saliva, 57 (34%) were willing to collect additional samples in the evening to determine the DLMO. A total number of 659 samples were available for analysis. Samples with undetectable melatonin levels (n = 178, 27.0%) were imputed with the lower limit of quantitation divided by two (5.0 pmol/l). The availability of melatonin profiles at T0 (100%) and T1 (93%) were similar between groups. It was possible to detect a DLMO for 48 profiles (84%) at T0 and 37 profiles (65%) at T1. Table S2.4 provides an overview of reasons for missing DLMO's and figure S2.2 shows the change in DLMO from pre- to post-intervention.

Table S2.4. Overview of reasons for missing DLMO's.

	Baseline	Post intervention
Profiles available	57 (100%)	53 (93%)
Deviation from protocol	4 (7%)	5 (9%)
Determined DLMO	48 (84)	37 (65)
Missing	9	20
No dynamic part	5	3
No base part		3
Insufficient number of data points	1	8
Time of collection not available/samples missing	3	6

Note: Deviation from protocol include drinking coffee or tea, eating chocolate, taking external melatonin, no closure of curtains, or withdrawal of consent to collect additional saliva in the evening.

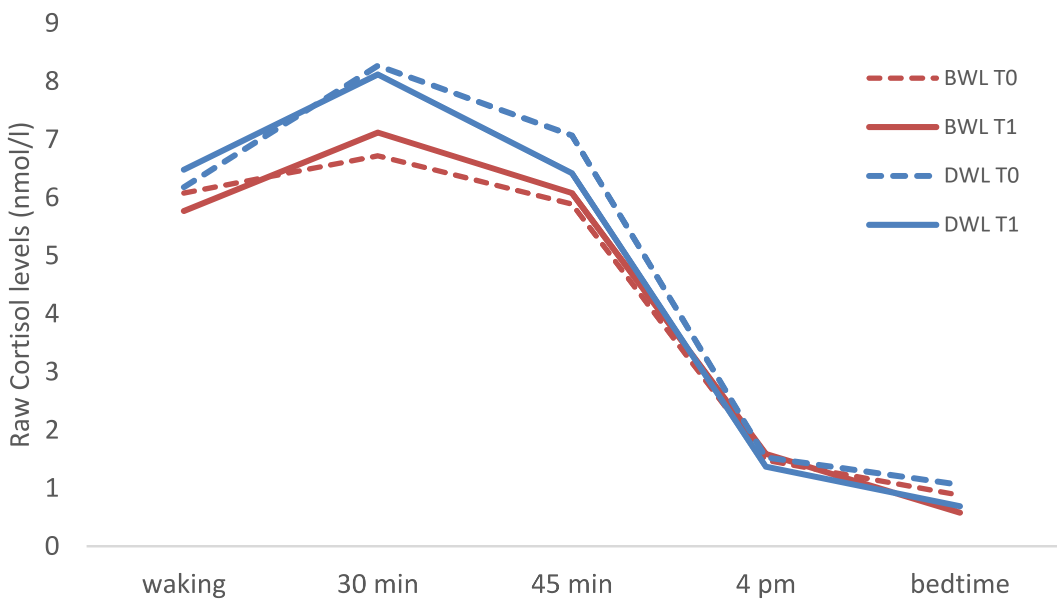

Figure S2.1. Average raw cortisol values for bright white light (BWL) and dim white light (DWL) conditions at each sampling time pre- and post-intervention.

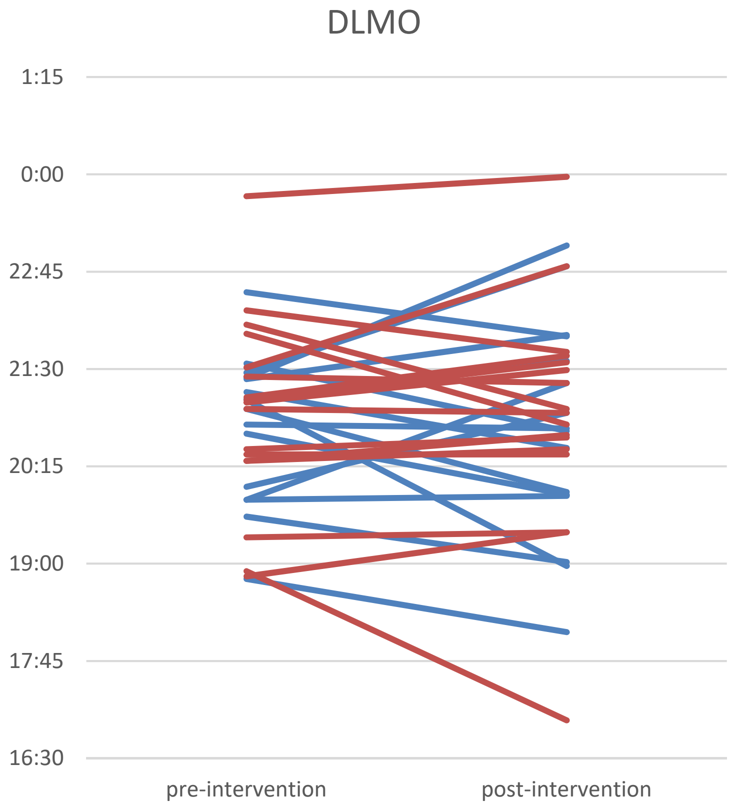

Figure S2.2. Overview of individual changes in DLMO in BWL (red) and DWL (blue) from pre- to post-intervention.

Supplementary material 3: Correction for missing data

Introduction

The rate of missing T1 questionnaires was significantly different between the intervention and control group (see the results section of the manuscript), with more missing data in the DWL group compared to the BWL group. This could lead to a potential bias in the results because the number of individuals that stopped prematurely with light therapy more often did not complete all assessments (Table S3.1). Consequently, fatigue levels might have been worse in the unobserved group compared to the observed group, which might affect the conclusions of the primary analyses.

Table S3.1. Missing data patterns (for groups that successfully completed light therapy or stopped light therapy

	n (%)	
Missing data pattern	Successfully completed	Premature stop
OOOO	136 (88.9)	3 (23.1)
OOOM / OOMO	8 (5.2)	1 (7.7)
OOMM	6 (3.9)	2 (23.1)
OMMM	3 (2.0)	5 (38.5)
MMMM	0 (0.0)	1 (7.7)

O=observed; M=missing

Method

To evaluate the possible effects of missing data on the study results, we used a pattern-mixture model in our primary growth curve model analyses. Supplementary material Table S3.2 shows the possible patterns of missing data. The pattern of missingsness was included as an independent variable in the growth curve model, as well as the interaction with group and time, to adjust for non-ignorable drop-out. A significant interaction between the missing data pattern and time or group indicates that having missing data predicted change in the dependent variables. In the analysis, we combined the patterns OOOM and OOMO as groups were small, and excluded the MMMM pattern (O=observed, M=Missing). The SPSS syntax for the dependent variable VAS-fatigue is as follows:

```
MIXED VAS with time_lin time_sq BY group marstat_cat patternmiss
  /CRITERIA=CIN(95) MXITER(100) MXSTEP(10) SCORING(1) SINGULAR(0.000000000001)
  HCONVERGE(0, ABSOLUTE) LCONVERGE(0, ABSOLUTE) PCONVERGE(0.000001,
ABSOLUTE)
  /FIXED time_lin time_sq group marstat_cat patternmiss time_lin*group time_sq*group
  time_lin*marstat_cat time_sq*marstat_cat group*marstat_cat time_lin*patternmiss
  time_sq*patternmiss group*patternmiss
  /METHOD ML
  /PRINT SOLUTION TESTCOV
  /RANDOM intercept time_lin | subject(ID) covtype(VC).
```

Where time_lin: 0 = T0; 1 = T1; 3.91 = T2; 9.44 = T3
time_sq: 0 = T0; 1 = T1; 15.29 = T2; 89.11 = T3
group: 0 = BWL; 1 = DWL
marstat_cat: 0 = single / widow; 1 = married or in a relationship
patternmiss: 1 = OOOM / OOMO; 2 = OOMM; 3= OMMM; 4 = OOOO.
Note: SPSS uses the category with the highest value as the reference group.

Table S3.2. Possible missing data patterns and their number of occurrence in the BWL and DWL group (Fisher's exact test p = .06).

					n (%)		
Missing data pattern	T0	T1	T2	T3	BWL	DWL	Total
OOOO	Observed	Observed	Observed	Observed	71 (85.5)	68 (81.9)	139 (83.7)
OOOM	Observed	Observed	Observed	Missing	1 (1.2)	3 (3.6)	4 (2.4)
OOMM	Observed	Observed	Missing	Missing	7 (8.4)	2 (2.4)	9 (5.4)
OMMM	Observed	Missing	Missing	Missing	1 (1.2)	7 (8.4)	8 (4.8)
MMMM	Missing	Missing	Missing	Missing	0 (0.0)	1 (1.2)	1 (0.6)
OOMO	Observed	Observed	Missing	Observed	3 (3.6)	2 (2.4)	5 (3.0)

Note: Data for BWL, DWL and total group are shown as No. (%).
T0 = baseline; T1 = post intervention; T2 = 3 months after light therapy; T3 = 9 months after light therapy

Results

The results of the growth curve model with adjustment for non-ignorable drop-out led to the same conclusions as the models without this adjustment: the significance levels were slightly different (ranging from -.21 to .11), and the effect sizes were slightly different (ranging from -.02 to .03; Supplementary material Table S3.3).

Discussion

The results of the pattern mixture model showed similar results compared to the non-adjusted models. Therefore, they support the robustness of the conclusions drawn from the primary analyses that BWL showed no superiority over DWL in the treatment of cancer-related fatigue, sleep quality, depression and anxiety.

Table S3.3. Mean values and standard deviations per time-point, and between-group difference for the growth curve models of primary and secondary measures corrected for non-ignorable dropout and marital status.

	T0 [a]		T1 [a]		T2 [a]		T3 [a]		Group difference linear time effect			Group difference quadratic time effect			ES [b]	
	n	M [SD]	n	M [SD]	n	M [SD]	n	M [SD]	B	SE	*p*	B	SE	*p*	T0-T1	T1-T3
PRIMARY OUTCOMES																
Fatigue																
VAS fatigue																
BWL	82	5.9 [1.8]	82	4.8 [1.9]	72	5.0 [2.1]	74	5.1 [2.3]	0.19	0.15	.21	-0.02	0.01	.31	0.18	0.08
DWL [c]	82	6.3 [1.4]	75	4.8 [1.9]	71	5.0 [1.7]	70	5.1 [2.1]								
MFI general fatigue																
BWL	83	15.6 [2.9]	82	13.2 [3.4]	72	13.6 [3.4]	74	13.8 [3.5]	0.08	0.24	.72	0.00	0.02	.98	0.05	0.17
DWL [c]	82	15.8 [2.5]	75	13.1 [3.3]	71	13.5 [3.4]	70	13.2 [3.6]								
Restrictions caused by fatigue (WSAS)																
BWL	83	20.7 [7.8]	82	17.4 [8.7]	72	17.5 [9.0]	74	17.3 [9.3]	-0.25	0.39	.52	0.03	0.04	.42	-0.15	0.15
DWL [c]	82	20.2 [8.5]	75	17.8 [8.8]	70	17.2 [8.8]	70	16.5 [9.2]								
SECONDARY OUTCOMES																
Sleep quality (PSQI) [d]																
BWL	83	7.5 [3.7]	82	6.3 [3.1]	72	6.6 [3.1]	74	6.4 [3.2]	0.02	0.21	.94	-0.01	0.02	.74	0.13	-0.16
DWL [c]	82	7.6 [3.6]	75	5.9 [3.1]	69	6.2 [3.1]	70	6.4 [3.0]								
Depression (CES-D)																
BWL	83	18.0 [4.6]	82	16.8 [4.7]	72	17.3 [4.6]	74	18.0 [5.5]	0.21	0.33	.52	-0.01	0.03	.87	0.28	0.22
DWL [c]	82	19.0 [5.0]	75	16.3 [4.5]	69	16.8 [3.9]	70	16.6 [4.7]								
Anxiety (STAI-6)																
BWL	83	39.6 [10.9]	82	37.4 [11.4]	72	38.7 [10.7]	74	39.0 [12.1]	0.62	0.70	.38	-0.06	0.07	.41	0.04	0.06
DWL [c]	82	39.9 [9.8]	75	37.2 [9.5]	69	36.7 [8.9]	69	38.1 [11.5]								
Quality of life (RAND-36)																
Physical functioning																
BWL	83	73.8 [20.5]	82	75.1 [20.6]	72	74.4 [21.5]	74	75.2 [24.0]	-0.43	0.24	.07				0.12	-0.26
DWL [c]	82	75.1 [19.5]	75	74.3 [21.7]	69	78.6 [19.5]	69	78.9 [18.9]								
Role functioning/physical																

BWL	83	33.7 [36.7]	82	49.1 [42.0]	72	38.2 [37.0]	74	50.7 [39.9]	-0.59	0.71	.41				0.14	-0.22
DWL [c]	82	39.0 [36.0]	75	49.7 [38.7]	69	55.1 [41.7]	69	59.1 [41.8]								
Role functioning/emotional																
BWL	83	71.9 [38.4]	82	74.8 [38.7]	72	74.5 [36.0]	74	70.7 [38.2]	0.11	0.65	.87				-0.04	-0.04
DWL [c]	82	72.4 [38.4]	75	76.9 [35.9]	69	73.9 [36.6]	69	74.4 [37.1]								
Energy																
BWL	83	42.9 [14.9]	82	50.4 [17.2]	72	49.9 [16.7]	73	50.7 [20.3]	-0.79	1.02	.44	0.05	0.10	.65	-0.03	-0.15
DWL [c]	82	41.2 [15.2]	75	49.7 [15.6]	69	50.4 [16.1]	69	51.9 [18.2]								
Emotional well-being																
BWL	83	73.0 [14.6]	82	75.0 [16.1]	72	72.7 [17.6]	73	71.2 [18.9]	-1.75	0.94	.06	0.14	0.09	.15	-0.18	-0.18
DWL [c]	82	69.9 [16.8]	75	75.0 [15.9]	69	75.6 [16.1]	69	74.6 [16.8]								
Social functioning																
BWL	83	59.6 [19.8]	82	68.0 [20.4]	72	66.8 [22.0]	74	69.6 [24.8]	0.97	1.29	.45	-0.10	0.13	.44	0.14	-0.10
DWL [c]	82	61.4 [20.2]	75	67.2 [19.1]	69	65.9 [19.4]	69	70.3 [21.8]								
Pain																
BWL	83	73.0 [25.3]	82	76.3 [22.3]	72	74.7 [25.1]	74	72.8 [25.7]	-0.92	1.25	.46	0.03	0.12	.79	0.07	-0.25
DWL [c]	82	70.2 [24.7]	75	71.3 [23.3]	69	74.4 [21.8]	69	74.8 [22.2]								
General health																
BWL	83	48.3 [17.3]	82	49.0 [17.4]	72	48.8 [20.6]	73	48.8 [21.3]	-0.32	0.25	.21				-0.01	-0.16
DWL [c]	82	50.2 [21.5]	75	51.9 [20.7]	69	53.6 [21.5]	69	54.5 [21.9]								

Notes:

Raw means and standard deviations are reported. Models were adjusted for non-ignorable drop-out and marital status

Abbreviations: **BWL** Bright white light; **CES-D** Center for Epidemiological Studies – Depression; **CWS** Cancer Worry Scale; **DWL** Dim white light; **FCS** Fatigue Catastrophizing Scale; **IS** Interdaily Stability; **IV** Intradaily Variability; **MFI** Multidimensional Fatigue inventory; **PSQI** Pittsburgh Sleep Quality Index; **RAND-36** RAND 36-item Health Survey; **SES** Self-efficacy Scale; **STAI-6** State Trait Anxiety Index – short form; **VAS fatigue** Visual Analogue Scale fatigue; **WSAS** Work and Social Adjustment Scale.

[a] **T0** baseline; **T1** post intervention; **T2** 3 months after light therapy; **T3** 9 months after light therapy

[b] The effect size was calculated based on the t test statistic (2*t)/(√df); small .20; moderate .50; large .80.

[c] DWL is reference group.

[d] Based on the AIC and BIC criteria, the model with the best fit excluded a random slope and included an autoregressive covariance structure.

Supplementary material 4: completion during Covid-19 restrictions.

Introduction

Thirty-three participants completed the 9 months follow-up assessment during the restrictions for the Covid-19 pandemic. This meant that these participants were encouraged to work from home and social activities were restricted. Consequently, sleep and work patterns could change and influence our results.

Method

To correct for this, we asked these participants to complete a survey on how the Covid-19 restrictions changed their sleep-wake cycle and whether it affected their fatigue level. We also performed the growth curve model with the exclusion of the final assessment of these participants.

Results

Results of the survey (Table S4.1) showed that the majority of the participants did not change their bedtimes (70%), nor did they experience a change in sleep quality (82%), although they reported changes in daily routine (88%). Sixty-three percent of the participants reported no effect of the Covid-19 restrictions on fatigue levels, 19% felt more fatigued and 11% felt less fatigue. The restrictions led to changes in the time that individuals spend outside. One third spend more time outside, 26% spend less time outside and 37% reported no change in time spend outside. Results of the growth curve model excluding the final assessments completed during Covid-19 restrictions led to similar conclusions as the unadjusted growth curve models (Supplementary material Table S4.2).

Discussion

Although the majority of the participants did not change their sleep behaviors because of the work and social activities restrictions due to Covid-19, the majority also mentioned that their daily activities and time spent outside changed. Yet, this did not affect self-reported sleep quality and fatigue levels. Moreover, the sensitivity analyses with the exclusion of the final assessment completed during Covid-19 restrictions showed similar results compared to the primary analyses.

Table S4.1. Overview of the self-reported influence of Covid-19 restrictions on sleep times, daily routine, and fatigue.

	Total (n = 26)
1a. Did your bedtimes change because of the Covid-19 restrictions?	
No	19 (70,4)
Yes	7 (25.9)
1b. If your bedtimes changes, how did they change?	
I go to bed earlier	3 (11.1)
I go to bed later	6 (22.2)
I get up earlier	2 (7.4)
I get up later	2 (7.4)
2. Do you feel like your sleep quality changed since the Covid-19 restrictions?	
No	22 (81.5)
Yes, I sleep better	2 (7.4)
Yes, I sleep worse	2 (7.4)
3. Is your daily routine more busy or more relaxed since the Covid-19 restrictions?	
Remained the same	6 (22.2)
Became more busy	3 (11.1)
Became more relaxed	7 (25.9)
My daily routine changed, but it wasn't more busy or relaxed	10 (37.0)
4. Do you spend more or less time outside since the Covid-19 restrictions?	
Remained the same	10 (37.0)
More time outside	9 (33.3)
Less time outside	7 (25.9)
5. Do you think that the Covid-19 restrictions had an effect on your fatigue?	
No	17 (63.0)
Yes, I feel more fatigued	5 (18.5)
Yes, I feel less fatigued	3 (11.1)

Note: Data are shown as No. (%).

Table S4.2. Mean values and standard deviations per time-point, and between-group difference for the growth curve models of primary and secondary measures excluding the final assessments completed during Covid-19 restrictions.

	T0 [a]		T1 [a]		T2 [a]		T3 [a]		Group difference linear time effect			Group difference quadratic time effect			ES [b]	
	n	M [SD]	n	M [SD]	n	M [SD]	n	M [SD]	B	SE	*p*	B	SE	*p*	T0-T1	T1-T3
PRIMARY OUTCOMES																
Fatigue																
VAS fatigue																
BWL	82	5.9 [1.8]	82	4.8 [1.9]	72	5.0 [2.1]	58	5.1 [2.3]	0.20	0.15	.17	-0.02	0.02	.19	0.18	-0.04
DWL [c]	82	6.3 [1.4]	75	4.8 [1.9]	71	5.0 [1.7]	53	5.2 [2.2]								
MFI general fatigue																
BWL	83	15.6 [2.9]	82	13.2 [3.4]	72	13.6 [3.4]	58	13.9 [3.5]	0.10	0.24	.68	0.00	0.02	.93	0.05	0.13
DWL [c]	82	15.8 [2.5]	75	13.1 [3.3]	71	13.5 [3.4]	53	13.6 [3.7]								
Restrictions caused by fatigue (WSAS)																
BWL	83	20.7 [7.8]	82	17.4 [8.7]	72	17.5 [9.0]	58	17.8 [9.5]	-0.23	0.39	.55	0.03	0.04	.41	-0.15	0.17
DWL [c]	82	20.2 [8.5]	75	17.8 [8.8]	70	17.2 [8.8]	53	16.8 [9.6]								
SECONDARY OUTCOMES																
Sleep quality (PSQI) [d]																
BWL	83	7.5 [3.7]	82	6.3 [3.1]	72	6.6 [3.1]	58	6.5 [3.3]	0.05	0.21	.81	-0.01	0.02	.74	0.13	-0.13
DWL [c]	82	7.6 [3.6]	75	5.9 [3.1]	69	6.2 [3.1]	53	6.5 [3.0]								
Depression (CES-D)																
BWL	83	18.0 [4.6]	82	16.8 [4.7]	72	17.3 [4.6]	58	18.0 [5.4]	0.28	0.33	.40	-0.01	0.03	.74	0.28	0.19
DWL [c]	82	19.0 [5.0]	75	16.3 [4.5]	69	16.8 [3.9]	53	16.9 [4.8]								
Anxiety (STAI-6)																
BWL	83	39.6 [10.9]	82	37.4 [11.4]	72	38.7 [10.7]	58	39.1 [11.9]	0.64	0.70	.36	-0.05	0.07	.51	0.04	0.12
DWL [c]	82	39.9 [9.8]	75	37.2 [9.5]	69	36.7 [8.9]	53	38.0 [12.0]								
Quality of life (RAND-36)																
Physical functioning																
BWL	83	73.8 [20.5]	82	75.1 [20.6]	72	74.4 [21.5]	58	72.4 [25.2]	-0.60	0.28	.03				0.12	-0.33
DWL [c]	82	75.1 [19.5]	75	74.3 [21.7]	69	78.6 [19.5]	53	78.3 [19.2]								

Role functioning/physical																
BWL	83	33.7 [36.7]	82	49.1 [42.0]	72	38.2 [37.0]	58	46.6 [41.2]	-0.85	0.81	.30				0.14	-0.27
DWL [c]	82	39.0 [36.0]	75	49.7 [38.7]	69	55.1 [41.7]	53	56.6 [43.9]								
Role functioning/emotional																
BWL	83	71.9 [38.4]	82	74.8 [38.7]	72	74.5 [36.0]	58	72.4 [37.5]	-0.15	0.74	.84				-0.04	-0.11
DWL [c]	82	72.4 [38.4]	75	76.9 [35.9]	69	73.9 [36.6]	53	75.5 [36.5]								
Energy																
BWL	83	42.9 [14.9]	82	50.4 [17.2]	72	49.9 [16.7]	57	50.9 [20.3]	-0.92	1.03	.37	0.06	0.10	.54	-0.03	-0.11
DWL [c]	82	41.2 [15.2]	75	49.7 [15.6]	69	50.4 [16.1]	53	49.7 [18.4]								
Emotional well-being																
BWL	83	73.0 [14.6]	82	75.0 [16.1]	72	72.7 [17.6]	57	72.1 [19.1]	-1.99	0.93	.03	0.17	0.09	.07	-0.18	-0.09
DWL [c]	82	69.9 [16.8]	75	75.0 [15.9]	69	75.6 [16.1]	53	72.2 [17.5]								
Social functioning																
BWL	83	59.6 [19.8]	82	68.0 [20.4]	72	66.8 [22.0]	58	68.3 [24.9]	0.87	1.27	.50	-0.07	0.13	.60	0.14	-0.02
DWL [c]	82	61.4 [20.2]	75	67.2 [19.1]	69	65.9 [19.4]	53	67.0 [22.6]								
Pain																
BWL	83	73.0 [25.3]	82	76.3 [22.3]	72	74.7 [25.1]	58	69.6 [26.8]	-0.66	1.26	.60	-0.01	0.13	.96	0.07	-0.29
DWL [c]	82	70.2 [24.7]	75	71.3 [23.3]	69	74.4 [21.8]	53	74.2 [22.9]								
General health																
BWL	83	48.3 [17.3]	82	49.0 [17.4]	72	48.8 [20.6]	58	46.1 [19.6]	-0.49	0.30	.11				-0.01	-0.22
DWL [c]	82	50.2 [21.5]	75	51.9 [20.7]	69	53.6 [21.5]	53	54.3 [21.8]								
Sleep actigraphy																
Sleep efficiency, %																
BWL	80	74.38 [7.71]	74	73.43 [7.75]	70	73.99 [7.68]	55	74.29 [8.95]	0.04	0.07	.62				-0.04	0.08
DWL [c]	77	74.04 [8.3]	73	73.48 [8.91]	68	74.23 [8.32]	53	73.35 [8.85]								
Mid-sleep time, hh:mm																
BWL	80	3:46 [0:46]	74	3:41 [0:47]	70	3:46 [0:46]	55	3:43 [0:41]	-20.58	42.00	.63				-0.05	0.00
DWL [c]	77	3:45 [0:52]	73	3:42 [0:52]	68	3:44 [0:47]	53	3:46 [0:57]								
Total sleep time, min																
BWL	80	6:23 [0:51]	74	6:16 [0:54]	70	6:18 [0:49]	55	6:16 [0:56]	90.68	119.86	.45	-13.04	12.21	.29	-0.06	-0.04
DWL [c]	77	6:20 [0:46]	73	6:14 [0:58]	68	6:19 [0:50]	53	6:20 [0:51]								

IS [e]																	
	BWL	79	0.76 [0.10]	73	0.75 [0.11]	68	0.75 [0.09]	54	0.77 [0.08]	0.00	0.01	1.00	0.00	0.00	.98	0.25	-0.07
	DWL [c]	77	0.76 [0.12]	72	0.73 [0.13]	67	0.75 [0.12]	53	0.75 [0.11]								
IV																	
	BWL	79	0.41 [0.10]	73	0.41 [0.12]	68	0.42 [0.11]	54	0.42 [0.10]	0.01	0.01	.37	0.00	0.00	.51	-0.16	0.16
	DWL [c]	77	0.40 [0.13]	72	0.41 [0.15]	67	0.40 [0.12]	53	0.41 [0.14]								

Notes:

Raw means and standard deviations are reported. Models were adjusted for marital status.

Abbreviations: **BWL** Bright white light; **CES-D** Center for Epidemiological Studies – Depression; **CWS** Cancer Worry Scale; **DWL** Dim white light; **FCS** Fatigue Catastrophizing Scale; **IS** Interdaily Stability; **IV** Intradaily Variability; **MFI** Multidimensional Fatigue inventory; **PSQI** Pittsburgh Sleep Quality Index; **RAND-36** RAND 36-item Health Survey; **SES** Self-efficacy Scale; **STAI-6** State Trait Anxiety Index – short form; **VAS fatigue** Visual Analogue Scale fatigue; **WSAS** Work and Social Adjustment Scale.

[a] **T0** baseline; **T1** post intervention; **T2** 3 months after light therapy; **T3** 9 months after light therapy

[b] The effect size was calculated based on the t test statistic (2*t)/(√df); small .20; moderate .50; large .80.

[c] DWL is reference group.

[d] Based on the AIC and BIC criteria, the model with the best fit excluded a random slope and included an autoregressive covariance structure.

[e] Due to convergence problems, an identity covariance matrix instead of variance components covariance structure was used.

Supplementary material 5: Additional tables for intention-to-treat, per protocol and sensitivity analyses.

Table S5.1. Mean values and standard deviations per time-point, and between-group differences of the primary and secondary measures for the growth curve models of the intention-to-treat analysis.

	T0 [a]		T1 [a]		T2 [a]		T3 [a]		Group difference linear time effect			Group difference quadratic time effect			ES [b]	
	n	M [SD]	n	M [SD]	n	M [SD]	n	M [SD]	B	SE	*p*	B	SE	*p*	T0-T1	T1-T3
PRIMARY OUTCOMES																
Fatigue																
VAS fatigue																
BWL	82	5.9 [1.8]	82	4.8 [1.9]	72	5.0 [2.1]	74	5.1 [2.3]	0.18	0.15	.23	-0.01	0.01	.32	0.18	0.08
DWL [c]	82	6.3 [1.4]	75	4.8 [1.9]	71	5.0 [1.7]	70	5.1 [2.1]								
MFI general fatigue																
BWL	83	15.6 [2.9]	82	13.2 [3.4]	72	13.6 [3.4]	74	13.8 [3.5]	0.08	0.24	.73	0.00	0.02	.98	0.05	0.17
DWL [c]	82	15.8 [2.5]	75	13.1 [3.3]	71	13.5 [3.4]	70	13.2 [3.6]								
Restrictions caused by fatigue (WSAS)																
BWL	83	20.7 [7.8]	82	17.4 [8.7]	72	17.5 [9.0]	74	17.3 [9.3]	-0.23	0.39	.56	0.03	0.04	.45	-0.15	0.15
DWL [c]	82	20.2 [8.5]	75	17.8 [8.8]	70	17.2 [8.8]	70	16.5 [9.2]								
SECONDARY OUTCOMES																
Sleep quality (PSQI) [d]																
BWL	83	7.5 [3.7]	82	6.3 [3.1]	72	6.6 [3.1]	74	6.4 [3.2]	0.07	0.21	.73	-0.01	0.02	.59	0.13	-0.16
DWL [c]	82	7.6 [3.6]	75	5.9 [3.1]	69	6.2 [3.1]	70	6.4 [3.0]								
Depression (CES-D)																
BWL	83	18.0 [4.6]	82	16.8 [4.7]	72	17.3 [4.6]	74	18.0 [5.5]	0.27	0.33	.41	-0.01	0.03	.75	0.28	0.22
DWL [c]	82	19.0 [5.0]	75	16.3 [4.5]	69	16.8 [3.9]	70	16.6 [4.7]								
Anxiety (STAI-6)																
BWL	83	39.6 [10.9]	82	37.4 [11.4]	72	38.7 [10.7]	74	39.0 [12.1]	0.72	0.69	.30	-0.07	0.07	.34	0.04	0.06
DWL [c]	82	39.9 [9.8]	75	37.2 [9.5]	69	36.7 [8.9]	69	38.1 [11.5]								
Quality of life (RAND-36)																
Physical functioning																

BWL	83	73.8 [20.5]	82	75.1 [20.6]	72	74.4 [21.5]	74	75.2 [24.0]	-0.42	0.23	.08				0.12	-0.26
DWL [c]	82	75.1 [19.5]	75	74.3 [21.7]	69	78.6 [19.5]	69	78.9 [18.9]								
Role limitations/physical																
BWL	83	33.7 [36.7]	82	49.1 [42.0]	72	38.2 [37.0]	74	50.7 [39.9]	-0.59	0.71	.41				0.14	-0.22
DWL [c]	82	39.0 [36.0]	75	49.7 [38.7]	69	55.1 [41.7]	69	59.1 [41.8]								
Role limitations/emotional																
BWL	83	71.9 [38.4]	82	74.8 [38.7]	72	74.5 [36.0]	74	70.7 [38.2]	0.02	0.64	.97				-0.04	-0.04
DWL [c]	82	72.4 [38.4]	75	76.9 [35.9]	69	73.9 [36.6]	69	74.4 [37.1]								
Energy																
BWL	83	42.9 [14.9]	82	50.4 [17.2]	72	49.9 [16.7]	73	50.7 [20.3]	-0.92	1.01	.37	0.05	0.10	.59	-0.03	-0.15
DWL [c]	82	41.2 [15.2]	75	49.7 [15.6]	69	50.4 [16.1]	69	51.9 [18.2]								
Emotional well-being																
BWL	83	73.0 [14.6]	82	75.0 [16.1]	72	72.7 [17.6]	73	71.2 [18.9]	-1.85	0.94	.05	0.14	0.09	.13	-0.18	-0.18
DWL [c]	82	69.9 [16.8]	75	75.0 [15.9]	69	75.6 [16.1]	69	74.6 [16.8]								
Social functioning																
BWL	83	59.6 [19.8]	82	68.0 [20.4]	72	66.8 [22.0]	74	69.6 [24.8]	1.05	1.28	.41	-0.11	0.13	.40	0.14	-0.10
DWL [c]	82	61.4 [20.2]	75	67.2 [19.1]	69	65.9 [19.4]	69	70.3 [21.8]								
Pain																
BWL	83	73.0 [25.3]	82	76.3 [22.3]	72	74.7 [25.1]	74	72.8 [25.7]	-0.74	1.25	.55	0.02	0.12	.90	0.07	-0.25
DWL [c]	82	70.2 [24.7]	75	71.3 [23.3]	69	74.4 [21.8]	69	74.8 [22.2]								
General health																
BWL	83	48.3 [17.3]	82	49.0 [17.4]	72	48.8 [20.6]	73	48.8 [21.3]	-0.35	0.26	.17				-0.01	-0.16
DWL [c]	82	50.2 [21.5]	75	51.9 [20.7]	69	53.6 [21.5]	69	54.5 [21.9]								
Actigraphy																
Sleep efficiency, %																
BWL	80	74.4 [7.7]	74	73.4 [7.8]	70	74.0 [7.7]	71	74.5 [8.3]	0.04	0.07	.55				-0.04	0.10
DWL [c]	77	74.0 [8.3]	73	73.5 [8.9]	68	74.2 [8.3]	69	73.8=9 [8.5]								
Mid-sleep time, hh:mm																
BWL	80	3:46 [0:46]	74	3:41 [0:47]	70	3:46 [0:46]	71	3:50 [0:47]	8.82	36.75	.81				-0.05	0.08
DWL [c]	77	3:45 [0:52]	73	3:42 [0:52]	68	3:44 [0:47]	69	3:44 [0:52]								
Total sleep time, hh:mm																

BWL	80	6:23 [0:51]	74	6:16 [0:54]	70	6:18 [0:49]	71	6:18 [0:54]	84.26	119.02	.48	-11.76	11.81	.32	-0.06	-0.05
DWL [c]	77	6:20 [0:46]	73	6:14 [0:58]	68	6:19 [0:50]	69	6:21 [0:52]								
IS																
BWL	79	0.76 [0.10]	73	0.75 [0.11]	68	0.75 [0.09]	69	0.77 [0.08]	0.00	0.01	.95	0.00	0.00	.99	0.25	-0.09
DWL [c]	77	0.76 [0.12]	72	0.73 [0.13]	67	0.75 [0.12]	69	0.75 [0.12]								
IV																
BWL	79	0.41 [0.10]	73	0.41 [0.12]	68	0.42 [0.11]	69	0.41 [0.09]	0.01	0.01	.28	0.00	0.00	.28	-0.16	0.03
DWL [c]	77	0.40 [0.13]	72	0.41 [0.15]	67	0.40 [0.12]	69	0.42 [0.17]								
Circadian rhythm																
Cortisol awakening response																
BWL	52	0.5 [3.4]	52	0.8 [3.6]					1.17	0.84	.17				0.27	
DWL [c]	57	1.7 [4.0]	52	1.1 [4.1]												
Diurnal cortisol slope																
BWL	63	-0.17 [0.08]	58	-0.18 [0.08]					0.00	0.02	.93				-0.06	
DWL [c]	66	-0.17 [0.09]	61	-0.17 [0.08]												
Total cortisol output																
BWL	60	41.2 [23.4]	48	41.3 [23.4]					7.52	5.16	.15				0.26	
DWL [c]	64	48.2 [26.0]	56	41.3 [19.2]												
DLMO, hh:mm																
BWL	24	20:39 [1:11]	18	20:46 [1:21]					-0.10	0.34	.77				-0.17	
DWL [c]	20	20:54 [1:20]	18	20:55 [1:27]												

Notes:

Raw means and standard deviations are reported. Models were adjusted for marital status

Abbreviations: **BWL** Bright white light; **CES-D** Center for Epidemiological Studies – Depression; **CWS** Cancer Worry Scale; **DLMO** Dim light melatonin onset; **DWL** Dim white light; **FCS** Fatigue Catastrophizing Scale; **IS** Interdaily Stability; **IV** Intradaily Variability; **MFI** Multidimensional Fatigue inventory; **PSQI** Pittsburgh Sleep Quality Index; **RAND-36** RAND 36-item Health Survey; **SES** Self-efficacy Scale; **STAI-6** State Trait Anxiety Index – short form; **VAS fatigue** Visual Analogue Scale fatigue; **WSAS** Work and Social Adjustment Scale.

[a] **T0** baseline; **T1** post intervention; **T2** 3 months after light therapy; **T3** 9 months after light therapy

[b] The effect size was calculated based on the t test statistic (2*t)/(√df); small .20; moderate .50; large .80.

[c] DWL is reference group.

[d] Based on the AIC and BIC criteria, the model with the best fit excluded a random slope and included an autoregressive covariance structure.

Table S5.2. Mean values and standard deviations per time-point and time effects for growth curve models of primary and secondary measures for the total sample.

	T0 [a]		T1 [a]		T2 [a]		T3 [a]		Linear time effect			Quadratic time effect			ES [b]	
	n	M [SD]	n	M [SD]	n	M [SD]	n	M [SD]	B	SE	*p*	B	SE	*p*	T0-T1	T1-T3
PRIMARY OUTCOMES																
Fatigue																
VAS fatigue	164	6.1 [1.6]	157	4.8 [1.9]	143	5.0 [1.9]	144	5.1 [2.2]	-0.40	0.07	<.001	0.03	0.01	<.001	-0.71	0.15
MFI general fatigue	165	15.7 [2.7]	157	13.1 [3.3]	143	13.6 [3.4]	144	13.5 [3.5]	-0.74	0.12	<.001	0.06	0.01	<.001	-0.81	0.13
Restrictions caused by fatigue (WSAS)	165	20.5 [8.2]	157	17.6 [8.7]	142	17.3 [8.9]	144	16.9 [9.2]	-1.07	0.19	<.001	0.08	0.02	<.001	-0.32	-0.07
SECONDARY OUTCOMES																
Sleep quality (PSQI) [c]	165	7.6 [3.7]	157	6.1 [3.1]	141	6.4 [3.1]	144	6.4 [3.1]	-0.40	0.10	<.001	0.03	0.01	.001	-0.44	0.10
Depression (CES-D)	165	18.5 [4.8]	157	16.6 [4.6]	141	17.1 [4.3]	144	17.3 [5.1]	-0.47	0.16	.004	0.04	0.02	.007	-0.41	0.16
Anxiety (STAI-6)	165	39.8 [10.4]	157	37.4 [10.5]	141	37.7 [9.9]	143	38.6 [11.8]	-0.79	0.34	.02	0.08	0.03	.02	-0.23	0.13
Quality of Life (RAND-36)																
Physical functioning	165	74.4 [20.0]	157	74.7 [21.1]	141	76.4 [20.6]	143	77.0 [21.7]	0.17	0.12	.14				0.01	0.07
Role functioning/physical	165	36.4 [36.4]	157	49.4 [40.3]	141	47.0 [40.0]	143	54.7 [40.9]	1.35	0.35	<.001				0.33	0.11
Role functioning/emotional	165	72.1 [38.3]	157	75.8 [37.3]	141	74.2 [36.2]	143	72.5 [37.6]	-0.08	0.32	.80				0.10	-0.08
Energy	165	42.1 [15.1]	157	50.1 [16.4]	141	50.1 [16.4]	142	51.3 [19.2]	2.86	0.50	<.001	-0.23	0.05	<.001	0.48	0.05
Emotional well-being	165	71.5 [15.8]	157	75.0 [15.9]	141	74.1 [16.9]	142	72.9 [17.9]	1.11	0.46	.02	-0.11	0.05	.01	0.21	-0.13
Social functioning	165	60.5 [19.9]	157	67.6 [19.8]	141	66.4 [20.7]	143	69.9 [23.3]	1.97	0.63	.002	-0.13	0.06	.03	0.35	0.09
Pain	165	71.6 [25.0]	157	73.9 [22.8]	141	74.5 [23.5]	143	73.8 [24.0]	1.06	0.61	.08	-0.10	0.06	.10	0.11	-0.04
General health	165	49.2 [19.4]	157	50.4 [19.0]	141	51.1 [21.1]	142	51.6 [21.7]	0.17	0.13	.19				0.05	0.04

Actigraphy																
Sleep efficiency, %	157	74.2 [8.0]	147	73.5 [8.3]	138	74.1 [8.0]	140	74.2 [8.4]	0.02	0.04	.55				-0.10	0.09
Mid-sleep time, hh:mm	157	3:45 [0:49]	147	3:41 [0:49]	138	3:45 [0:46]	140	3:47 [0:50]	31.41	18.01	.08				-0.02	0.11
Total sleep time, hh:mm	157	6:21 [0:49]	147	6:15 [0:55]	138	6:19 [0:49]	140	6:19 [0:53]	-58.72	58.94	.32	6.50	5.85	.27	-0.11	0.08
IS	156	0.76 [0.11]	145	0.74 [0.12]	135	0.75 [0.10]	138	0.76 [0.10]	-0.01	0.00	.07	0.00	0.00	.04	-0.24	0.22
IV	156	0.41 [0.12]	145	0.41 [0.14]	135	0.41 [0.12]	138	0.41 [0.14]	0.00	0.00	.55	0.00	0.00	.78	0.02	0.06
Circadian rhythm																
Cortisol awakening response	109	1.1 [3.8]	104	0.9 [3.9]					-0.27	0.42	.52				-0.07	
Diurnal cortisol slope	129	-0.17 [0.08]	119	-0.18 [0.08]					-0.01	0.01	.49				-0.08	
Total cortisol output	124	44.8 [24.9]	104	41.3 [21.1]					-3.04	2.55	.24				-0.13	
DLMO, hh:mm	44	20:46 [1:15]	36	20:50 [1:23]					-0.01	0.16	.94				-0.01	

Notes:

Raw means and standard deviations are reported.

Abbreviations: **CES-D** Center for Epidemiological Studies – Depression; **CWS** Cancer Worry Scale; **DLMO** Dim light melatonin onset; **FCS** Fatigue Catastrophizing Scale; **IS** Interdaily Stability; **IV** Intradaily Variability; **MFI** Multidimensional Fatigue inventory; **PSQI** Pittsburgh Sleep Quality Index; **RAND-36** RAND 36-item Health Survey; **SES** Self-efficacy Scale; **STAI-6** State Trait Anxiety Index – short form; **VAS fatigue** Visual Analogue Scale fatigue; **WSAS** Work and Social Adjustment Scale.

[a] **T0** baseline; **T1** post intervention; **T2** 3 months after light therapy; **T3** 9 months after light therapy

[b] The effect size was calculated based on the t test statistic (2*t)/(√df); small .20; moderate .50; large .80.

[c] Based on the AIC and BIC criteria, the model with the best fit excluded a random slope and included an autoregressive covariance structure.

Table S5.3: Mean values and standard deviations per time-point, and between-group differences of the primary and secondary measures for the growth curve models for individuals who adhered to 25 days of light therapy.

	T0 [a]		T1 [a]		T2 [a]		T3 [a]		Group difference linear time effect			Group difference quadratic time effect			ES [b]	
	n	M [SD]	n	M [SD]	n	M [SD]	n	M [SD]	B	SE	*p*	B	SE	*p*	T0-T1	T1-T3
PRIMARY OUTCOMES																
Fatigue																
VAS fatigue																
BWL	32	6.3 [1.9]	33	5.0 [1.9]	31	5.2 [2.3]	32	5.4 [2.3]	0.18	0.24	.44	-0.02	0.02	.45	0.11	-0.06
DWL [c]	28	6.3 [1.8]	28	4.8 [2.0]	27	4.8 [1.8]	28	5.3 [2.4]								
MFI general fatigue																
BWL	33	15.7 [3.1]	33	13.7 [3.4]	31	13.9 [4.1]	32	14.3 [3.5]	0.34	0.36	.34	-0.03	0.04	.41	0.40	-0.15
DWL [c]	28	16.0 [2.3]	28	12.6 [2.9]	27	13.4 [3.4]	28	13.6 [3.6]								
Restrictions caused by fatigue (WSAS)																
BWL	33	23.6 [8.4]	33	19.0 [8.9]	31	20.2 [9.1]	32	19.9 [8.4]	0.46	0.64	.48	-0.04	0.06	.51	-0.20	0.14
DWL [c]	28	22.1 [8.5]	28	19.3 [9.2]	26	18.4 [10.0]	28	18.7 [10.7]								
SECONDARY OUTCOMES																
Sleep quality (PSQI) [d]																
BWL	33	8.2 [4.0]	33	6.4 [2.9]	31	6.2 [3.1]	32	6.5 [3.1]	0.06	0.35	.86	-0.02	0.03	.57	-0.13	-0.21
DWL [c]	28	7.0 [3.7]	28	5.6 [2.5]	26	5.3 [2.8]	28	6.4 [2.8]								
Depression (CES-D)																
BWL	33	19.2 [4.3]	33	16.7 [4.3]	31	17.4 [4.1]	32	18.1 [5.8]	-0.29	0.50	.56	0.03	0.05	.58	-0.24	0.14
DWL [c]	28	17.0 [4.1]	28	15.5 [3.4]	26	16.1 [4.7]	28	16.4 [5.6]								
Anxiety (STAI-6)																
BWL	33	41.2 [11.8]	33	36.1 [10.6]	31	38.3 [10.8]	32	39.2 [11.2]	-0.14	1.15	.90	0.00	0.11	.99	-0.30	0.06

DWL [c]	28	36.7 [8.0]	28	34.5 [7.6]	26	34.7 [8.6]	27	36.5 [10.4]								
Quality of life (RAND-36)																
Physical functioning [e]																
BWL	33	67.7 [22.6]	33	70.0 [22.9]	31	66.6 [22.7]	32	68.8 [25.9]	-0.12	0.90	.89				0.31	-0.26
DWL [c]	28	74.8 [20.5]	28	70.2 [22.2]	26	74.2 [19.5]	27	74.6 [21.1]								
Role functioning/physical																
BWL	33	28.0 [34.7]	33	46.2 [40.6]	31	32.3 [37.7]	32	41.4 [41.5]	-0.14	1.14	.91				0.33	-0.21
DWL [c]	28	42.0 [38.5]	28	47.3 [35.6]	26	51.9 [44.1]	27	50.9 [43.6]								
Role functioning/emotional																
BWL	33	74.7 [37.3]	33	79.8 [36.3]	31	73.1 [35.9]	32	76.0 [36.2]	0.60	0.99	.55				0.05	0.08
DWL [c]	28	86.9 [24.6]	28	90.5 [25.4]	26	87.2 [23.2]	27	84.0 [31.2]								
Energy																
BWL	33	41.1 [15.7]	33	50.0 [15.8]	31	47.9 [17.6]	31	48.5 [22.5]	-2.57	1.53	.10	0.23	0.15	.13	0.07	-0.20
DWL [c]	28	40.2 [15.2]	28	47.9 [13.8]	26	51.9 [15.7]	27	49.6 [19.5]								
Emotional well-being																
BWL	33	73.0 [15.0]	33	78.1 [13.4]	31	73.5 [19.0]	31	71.9 [19.4]	-3.55	1.43	.01	0.35	0.14	.02	-0.19	0.00
DWL [c]	28	72.1 [14.5]	28	80.3 [12.7]	26	80.9 [13.8]	27	74.4 [18.0]								
Social functioning																
BWL	33	59.1 [20.6]	33	67.4 [22.1]	31	67.7 [23.9]	32	65.6 [27.9]	0.48	2.15	.82	-0.06	0.21	.78	0.05	-0.06
DWL [c]	28	61.2 [21.3]	28	68.3 [20.5]	26	65.9 [21.1]	27	67.6 [27.6]								
Pain																
BWL	33	69.7 [22.7]	33	69.5 [24.2]	31	69.7 [25.2]	32	68.9 [26.9]	-1.46	1.99	.47	0.11	0.20	.59	-0.07	-0.08
DWL [c]	28	67.6 [27.4]	28	69.1 [23.4]	26	70.5 [23.3]	27	70.7 [26.2]								
General health																
BWL	33	47.1 [16.7]	33	46.7 [17.3]	31	45.8 [21.2]	31	44.4 [20.9]	-0.25	0.39	.52				-0.25	0.03

DWL [c]	28	50 [19.0]	28	54.3 [17.9]	26	55.2 [19.1]	27	51.9 [21.2]								
Sleep (actigraphy)																
Sleep efficiency, %																
BWL	32	74.0 [8.7]	32	73.7 [8.6]	30	74.1 [8.9]	30	75.8 [8.8]	0.35	0.11	.002				-0.02	0.40
DWL [c]	28	75.6 [7.6]	28	75.6 [8.4]	26	75.2 [8.1]	27	74.0 [9.4]								
Mid-sleep time, hh:mm																
BWL	32	3:36 [0:42]	32	3:36 [0:49]	30	3:41 [0:42]	30	3:40 [0:41]	16.42	57.56	.78				0.01	0.07
DWL [c]	28	3:37 [0:41]	28	3:36 [0:42]	26	3:43 [0:49]	27	3:38 [0:55]								
Total sleep time, min																
BWL	32	6:18 [0:56]	32	6:13 [0:57]	30	6:23 [0:51]	30	6:24 [0:55]	321.82	172.87	.07	-21.16	17.20	.22	-0.12	0.35
DWL [c]	28	6:18 [0:49]	28	6:20 [1:00]	26	6:19 [0:53]	27	6:12 [0:58]								
IS																
BWL	32	0.76 [0.12]	32	0.73 [0.11]	30	0.75 [0.08]	30	0.78 [0.07]	0.00	0.01	.80	0.00	0.00	.91	0.02	0.33
DWL [c]	28	0.79 [0.09]	28	0.76 [0.12]	26	0.77 [0.11]	27	0.77 [0.14]								
IV																
BWL	32	0.42 [0.11]	32	0.41 [0.14]	30	0.43 [0.13]	30	0.40 [0.08]	0.01	0.01	.58	0.00	0.00	.42	-0.12	-0.25
DWL [c]	28	0.39 [0.08]	28	0.40 [0.10]	26	0.40 [0.09]	27	0.42 [0.21]								
Circadian rhythm																
Cortisol Awakening Response																
BWL	22	-0.4 [2.5]	25	0.6 [3.6]					2.64	1.33	.05				0.67	
DWL [c]	19	2.4 [4.5]	20	0.9 [4.0]												
Diurnal cortisol slope																
BWL	26	-0.15 [0.06]	25	-0.19 [0.08]					-0.02	0.03	.52				-0.29	
DWL [c]	21	-0.18 [0.08]	19	-0.20 [0.08]												
Total cortisol output																
BWL	26	36.2 [16.9]	21	37.8 [22.0]					5.15	8.12	.53				0.29	
DWL [c]	20	50.1 [20.7]	17	46.1 [22.1]												
DLMO, hh:mm																

BWL	12	20:23 [1:17]	9	20:14 [1:11]					-0.06	0.30	.86		0.12
DWL [c]	8	20:36 [1:26]	8	20:09 [1:31]									

Notes:

Raw means and standard deviations are reported. Models were adjusted for marital status

Abbreviations: **BWL** Bright white light; **CES-D** Center for Epidemiological Studies – Depression; **CWS** Cancer Worry Scale; **DLMO** Dim light melatonin onset; **DWL** Dim white light; **FCS** Fatigue Catastrophizing Scale; **IS** Interdaily Stability; **IV** Intradaily Variability; **MFI** Multidimensional Fatigue inventory; **PSQI** Pittsburgh Sleep Quality Index; **RAND-36** RAND 36-item Health Survey; **SES** Self-efficacy Scale; **STAI-6** State Trait Anxiety Index – short form; **VAS fatigue** Visual Analogue Scale fatigue; **WSAS** Work and Social Adjustment Scale.

[a] **T0** baseline; **T1** post intervention; **T2** 3 months after light therapy; **T3** 9 months after light therapy

[b] The effect size was calculated based on the t test statistic (2*t)/(√df); small .20; moderate .50; large .80.

[c] DWL is reference group.

[d] Based on the AIC and BIC criteria, the model with the best fit excluded a random slope and included an autoregressive covariance structure.

[e] identity covariance matrix

Table S5.4: Mean values and standard deviations per time-point, and between-group differences of the primary and secondary measures for the growth curve models for individuals who used the Luminette glasses.

	T0 [a]		T1 [a]		T2 [a]		T3 [a]		Group difference linear time effect			Group difference quadratic time effect			ES [b]	
	n	M [SD]	n	M [SD]	n	M [SD]	n	M [SD]	B	SE	*p*	B	SE	*p*	T0-T1	T1-T3
PRIMARY OUTCOMES																
Fatigue																
VAS fatigue																
BWL	65	5.9 [1.8]	64	4.7 [2.0]	55	4.8 [2.1]	57	5.0 [2.3]	-0.05	0.17	.78	0.01	0.02	.68	0.08	0.05
DWL [c]	62	6.3 [1.5]	58	4.9 [1.9]	55	5.3 [1.5]	55	5.2 [1.9]								
MFI general fatigue																
BWL	65	15.5 [2.8]	64	13.2 [3.4]	55	13.5 [3.2]	57	13.8 [3.4]	-0.17	0.27	.54	0.03	0.03	.36	0.13	0.08
DWL [c]	62	15.7 [2.5]	58	12.9 [3.5]	55	14.0 [3.3]	55	13.3 [3.4]								
Restrictions caused by fatigue (WSAS)																
BWL	65	20.8 [7.3]	64	17.1 [8.3]	55	17.2 [8.6]	57	17.1 [9.1]	-0.45	0.47	.34	0.05	0.05	.25	-0.20	0.16
DWL [c]	62	20.2 [8.5]	58	17.8 [9.1]	54	17.5 [8.5]	55	16.3 [8.7]								
SECONDARY OUTCOMES																
Sleep quality (PSQI) [d]																
BWL	65	7.7 [3.8]	64	6.2 [3.0]	55	6.6 [3.0]	57	6.5 [3.1]	-0.16	0.23	.49	0.01	0.02	.54	-0.01	-0.02
DWL [c]	62	7.2 [3.6]	58	5.5 [2.8]	53	6.2 [3.2]	55	5.9 [2.9]								
Depression (CES-D) [e]																
BWL	65	18.0 [4.7]	64	16.8 [4.7]	55	17.6 [4.8]	57	17.9 [5.4]	0.55	0.47	.24	-0.04	0.05	.43	0.29	0.25
DWL [c]	62	19.0 [4.9]	58	16.3 [4.7]	53	16.8 [4.0]	55	16.4 [4.5]								
Anxiety (STAI-6)																
BWL	65	39.5 [10.8]	64	38.5 [11.8]	55	39.5 [11.1]	57	38.9 [12.1]	0.96	0.77	.22	-0.09	0.08	.26	0.12	0.01
DWL [c]	62	39.8 [9.4]	58	37.4 [10.2]	53	36.8 [8.5]	54	37.6 [11.2]								
Quality of life (RAND-36)																
Physical functioning																
BWL	65	74.2 [20.3]	64	75.9 [19.9]	55	75.8 [21.4]	57	76.7 [23.1]	-0.32	0.26	.23				0.17	-0.25

DWL [c]	62	76.5 [17.3]	58	75.6 [19.4]	53	78.2 [18.6]	54	80.3 [17.0]								
Role functioning/physical																
BWL	65	33.8 [37.6]	64	50.4 [41.9]	55	38.2 [36.3]	57	50.4 [40.2]	-0.65	0.84	.44				0.20	-0.26
DWL [c]	62	41.5 [35.6]	58	50.9 [37.7]	53	52.8 [41.2]	54	60.2 [41.9]								
Role functioning/emotional [e]																
BWL	65	71.8 [38.3]	64	74.0 [39.2]	55	73.3 [36.5]	57	67.3 [39.6]	-0.80	1.05	.45				-0.08	-0.15
DWL [c]	62	71.5 [39.0]	58	77.0 [34.9]	53	73.6 [35.4]	54	75.9 [35.1]								
Energy																
BWL	65	42.0 [15.0]	64	49.8 [17.5]	55	49.8 [16.9]	57	50.4 [19.6]	0.02	1.17	.98	-0.04	0.12	.70	-0.02	-0.15
DWL [c]	62	41.4 [14.9]	58	50.0 [15.7]	53	49.4 [16.3]	54	53.1 [18.1]								
Emotional well-being																
BWL	65	71.6 [15.1]	64	74.7 [16.3]	55	70.9 [18.4]	57	70.2 [18.7]	-1.31	1.06	.22	0.08	0.11	.47	-0.04	-0.32
DWL [c]	62	70.5 [16.9]	58	74.6 [16.3]	53	75.1 [15.5]	54	76.1 [15.4]								
Social functioning																
BWL	65	58.8 [19.0]	64	67.8 [20.0]	55	66.4 [19.8]	57	69.5 [24.4]	3.16	1.38	.02	-0.33	0.14	.02	0.23	-0.19
DWL [c]	62	62.5 [18.4]	58	67.5 [19.7]	53	63.0 [19.3]	54	72.9 [20.0]								
Pain																
BWL	65	72.9 [25.8]	64	75.4 [21.5]	55	74.5 [24.9]	57	72.6 [24.5]	-0.65	1.46	.66	0.00	0.15	.98	0.03	-0.23
DWL [c]	62	70.4 [24.4]	58	71.7 [23.0]	53	73.8 [22.2]	54	74.4 [21.6]								
General health																
BWL	65	48.1 [17.6]	64	49.2 [17.0]	55	49.3 [20.5]	57	49.3 [19.9]	-0.39	0.29	.18				-0.02	-0.16
DWL [c]	62	50.5 [19.2]	58	52.5 [20.2]	53	52.2 [21.3]	54	54.5 [22.1]								
Sleep (actigraphy)																
Sleep efficiency, %																
BWL	63	74.90 [7.21]	57	73.68 [7.43]	53	74.39 [7.10]	54	74.51 [7.68]	-0.03	0.08	.76				-0.10	0.07
DWL [c]	59	74.37 [6.71]	56	73.72 [7.68]	52	74.74 [7.26]	55	74.43 [7.71]								
Mid-sleep time, hh:mm																
BWL	63	3:49 [0:48]	57	3:41 [0:48]	53	3:48 [0:50]	54	3:53 [0:51]	-2.57	43.53	.95				-0.14	0.09
DWL [c]	59	3:37 [0:40]	56	3:38 [0:48]	52	3:42 [0:42]	55	3:43 [0:50]								
Total sleep time, min																
BWL	63	6:23 [0:50]	57	6:14 [0:52]	53	6:20 [0:48]	54	6:17 [0:54]	48.63	134.39	.72	-8.74	13.31	.51	-0.11	-0.05

DWL [c]	59	6:21 [0:42]	56	6:16 [0:51]	52	6:21 [0:47]	55	6:21 [0:51]								
IS																
BWL	62	0.76 [0.10]	56	0.74 [0.11]	51	0.74 [0.09]	52	0.76 [0.09]	0.00	0.01	.94	0.00	0.00	.92	0.17	-0.07
DWL [c]	59	0.77 [0.10]	56	0.73 [0.14]	52	0.74 [0.11]	55	0.75 [0.12]								
IV																
BWL	62	0.41 [0.09]	56	0.40 [0.12]	51	0.42 [0.11]	52	0.40 [0.09]	0.01	0.01	.48	0.00	0.00	.44	-0.18	-0.04
DWL [c]	59	0.39 [0.11]	56	0.41 [0.12]	52	0.40 [0.11]	55	0.42 [0.17]								
Circadian rhythm																
Cortisol Awakening Response																
BWL	42	0.4 [3.6]	41	1.1 [3.5]					1.25	0.95	.19				0.28	
DWL [c]	44	1.8 [3.9]	39	1.3 [4.1]												
Diurnal cortisol slope																
BWL	49	-0.16 [0.08]	43	-0.16 [0.08]					-0.01	0.02	.81				-0.10	
DWL [c]	48	-0.17 [0.08]	44	-0.16 [0.07]												
Total cortisol output																
BWL	46	42.0 [25.5]	38	40.0 [23.7]					5.65	5.63	.32				0.18	
DWL [c]	47	49.7 [25.1]	41	41.9 [19.1]												
DLMO, hh:mm																
BWL	15	20:24 [1:04]	9	20:53 [1:32]					0.13	0.55	.82				0.17	
DWL [c]	8	19:54 [0:53]	10	20:18 [1:25]												

Notes:

Raw means and standard deviations are reported. Models were adjusted for marital status

Abbreviations: **BWL** Bright white light; **CES-D** Center for Epidemiological Studies – Depression; **CWS** Cancer Worry Scale; **DLMO** Dim light melatonin onset; **DWL** Dim white light; **FCS** Fatigue Catastrophizing Scale; **IS** Interdaily Stability; **IV** Intradaily Variability; **MFI** Multidimensional Fatigue inventory; **PSQI** Pittsburgh Sleep Quality Index; **RAND-36** RAND 36-item Health Survey; **SES** Self-efficacy Scale; **STAI-6** State Trait Anxiety Index – short form; **VAS fatigue** Visual Analogue Scale fatigue; **WSAS** Work and Social Adjustment Scale.

[a] **T0** baseline; **T1** post intervention; **T2** 3 months after light therapy; **T3** 9 months after light therapy

[b] The effect size was calculated based on the t test statistic (2*t)/(√df); small .20; moderate .50; large .80.

[c] DWL is reference group.

[d] Based on the AIC and BIC criteria, the model with the best fit excluded a random slope and included an autoregressive covariance structure.

[e] identity covariance structure

Table S5.5. Mean values and standard deviations per time-point, and between-group differences of the primary and secondary measures for the growth curve models for individuals who used the light therapy during autumn or winter.

	T0 [a]		T1 [a]		T2 [a]		T3 [a]		Group difference linear time effect			Group difference quadratic time effect			ES [b]	
	n	M [SD]	n	M [SD]	n	M [SD]	n	M [SD]	B	SE	*p*	B	SE	*p*	T0-T1	T1-T3
PRIMARY OUTCOMES																
Fatigue																
VAS fatigue																
BWL	45	5.8 [1.7]	46	4.6 [1.9]	39	4.8 [2.1]	42	4.8 [2.3]	0.22	0.21	.30	-0.02	0.02	.24	0.32	-0.34
DWL [c]	43	6.6 [1.5]	41	4.8 [2.0]	40	5.2 [1.5]	39	5.7 [2.0]								
MFI general fatigue																
BWL	46	15.6 [2.6]	46	12.9 [3.5]	39	13.4 [3.3]	42	13.3 [3.3]	-0.03	0.32	.93	0.01	0.03	.86	0.09	-0.06
DWL [c]	43	16.6 [2.5]	41	13.5 [3.3]	40	14.3 [3.1]	39	14.2 [3.7]								
Restrictions caused by fatigue (WSAS)																
BWL	46	20.3 [7.5]	46	16.5 [7.7]	39	17.3 [8.7]	42	16.8 [9.3]	-0.15	0.54	.78	0.01	0.05	.80	-0.08	-0.01
DWL [c]	43	22.4 [8.5]	41	19.0 [9.3]	39	19.5 [9.4]	39	19.2 [9.3]								
SECONDARY OUTCOMES																
Sleep quality (PSQI) [d]																
BWL	46	7.1 [3.6]	46	5.9 [2.9]	39	6.3 [3.1]	42	6.2 [3.0]	0.10	0.27	.72	-0.01	0.03	.67	0.22	-0.21
DWL [c]	43	7.3 [3.3]	41	5.3 [2.2]	39	6.1 [3.0]	39	6.3 [2.9]								
Depression (CES-D)																
BWL	46	17.6 [4.2]	46	16.5 [3.7]	39	16.7 [4.3]	42	17.1 [4.9]	-0.02	0.45	.97	0.00	0.45	.94	0.40	-0.14
DWL [c]	43	18.9 [5.4]	41	15.9 [4.6]	39	17.2 [3.9]	39	17.4 [5.5]								
Anxiety (STAI-6)																
BWL	46	37.9 [10.1]	46	36.4 [10.4]	39	36.7 [10.3]	42	37.9 [9.2]	0.66	0.97	.50	-0.05	0.10	.60	0.22	-0.01
DWL [c]	43	40.9 [8.3]	41	37.2 [9.1]	39	37.4 [9.5]	38	38.4 [12.0]								
Quality of life (RAND-36)																
Physical functioning																
BWL	46	74.8 [19.7]	46	74.5 [19.6]	39	74.4 [20.4]	42	74.4 [24.2]	-0.48	0.33	.15				0.01	-0.21

DWL [c]	43	74.0 [19.9]	41	74.1 [23.3]	39	76.0 [21.1]	38	77.6 [19.2]								
Role functioning/physical																
BWL	46	37.0 [33.2]	46	52.7 [41.2]	39	39.1 [38.0]	42	49.4 [41.1]	-0.67	1.00	.51				0.23	-0.22
DWL [c]	43	39.0 [39.5]	41	46.3 [39.7]	39	50.6 [41.9]	38	50.7 [43.7]								
Role functioning/emotional																
BWL	46	78.3 [34.6]	46	79.7 [36.2]	39	80.3 [29.3]	42	73.8 [37.2]	-0.23	0.91	.81				-0.20	-0.06
DWL [c]	43	68.2 [39.1]	41	78.0 [34.6]	39	70.1 [37.3]	38	73.7 [38.1]								
Energy																
BWL	46	46.1 [11.8]	46	53.8 [15.5]	39	50.9 [15.0]	41	54.4 [18.3]	-2.12	1.33	.11	0.19	0.13	.15	-0.17	0.03
DWL [c]	43	36.6 [14.3]	41	47.6 [17.4]	39	45.9 [15.0]	38	47.2 [18.2]								
Emotional well-being																
BWL	46	76.2 [13.7]	46	76.6 [15.5]	39	75.2 [16.9]	41	75.3 [17.2]	-2.57	1.18	.03	0.24	0.12	.05	-0.41	0.07
DWL [c]	43	67.1 [16.2]	41	74.4 [16.6]	39	75.0 [16.3]	38	72.5 [16.9]								
Social functioning																
BWL	46	62.5 [16.2]	46	71.2 [16.6]	39	69.2 [18.8]	42	72.3 [21.5]	0.51	1.65	.76	-0.03	0.16	.85	0.08	0.03
DWL [c]	43	56.4 [19.0]	41	63.7 [19.1]	39	61.5 [19.3]	38	64.1 [22.7]								
Pain																
BWL	46	70.7 [24.8]	46	78.6 [22.5]	39	73.3 [27.1]	42	72.7 [26.4]	0.23	1.64	.89	-0.07	0.16	.67	0.27	-0.25
DWL [c]	43	70.5 [26.1]	41	71.5 [23.4]	39	72.5 [22.6]	38	72.0 [24.2]								
General health																
BWL	46	46.5 [15.5]	46	48.6 [17.9]	39	46.7 [19.7]	41	46.0 [20.2]	-0.61	0.37	.10				-0.06	-0.26
DWL [c]	43	47.8 [20.2]	41	51.6 [19.2]	39	52.9 [19.3]	38	53.8 [21.1]								
Sleep (actigraphy)																
Sleep efficiency. %																
BWL	44	75.00 [8.25]	41	74.27 [7.89]	39	75.04 [8.26]	40	75.63 [9.05]	0.06	0.09	.53				-0.01	0.11
DWL [c]	42	73.83 [7.09]	41	73.46 [7.71]	39	74.27 [7.52]	39	73.76 [7.51]								
Mid-sleep time. hh:mm																
BWL	44	3:42 [0:43]	41	3:36 [0:46]	39	3:43 [0:48]	40	3:44 [0:41]	-14.15	48.95	.77				-0.06	0.04
DWL [c]	42	3:54 [0:59]	41	3:50 [1:01]	39	3:50 [0:48]	39	3:53 [0:55]								
Total sleep time, min																
BWL	44	6:26 [0:54]	41	6:24 [0:52]	39	6:21 [0:55]	40	6:17 [0:57]	141.36	151.97	.35	-18.78	15.08	.22	-0.02	-0.07

DWL [c]	42	6:25 [0:37]	41	6:24 [0:48]	39	6:18 [0:44]	39	6:22 [0:49]								
IS [e]																
BWL	43	0.77 [0.10]	40	0.77 [0.09]	38	0.75 [0.09]	39	0.77 [0.08]	-0.01	0.01	.56	0.00	0.00	.70	0.39	-0.41
DWL [c]	42	0.76 [0.12]	41	0.72 [0.14]	39	0.76 [0.11]	39	0.77 [0.10]								
IV																
BWL	43	0.42 [0.11]	40	0.41 [0.11]	38	0.42 [0.13]	39	0.41 [0.10]	0.01	0.01	.40	0.00	0.00	.42	-0.21	0.12
DWL [c]	42	0.40 [0.12]	41	0.42 [0.13]	39	0.39 [0.11]	39	0.41 [0.14]								
Circadian rhythm																
Cortisol Awakening Response																
BWL	27	-0.2 [3.1]	29	0.4 [3.8]					1.37	1.32	.30				0.34	
DWL [c]	31	1.5 [4.6]	29	0.7 [4.3]												
Diurnal cortisol slope																
BWL	35	-0.17 [0.07]	32	-0.19 [0.09]					-0.01	0.03	.76				-0.13	
DWL [c]	26	-0.18 [0.08]	24	-0.19 [0.08]												
Total cortisol output																
BWL	35	37.4 [16.0]	28	40.3 [25.5]					10.21	7.22	.16				0.53	
DWL [c]	26	47.3 [21.2]	21	40.8 [17.6]												
DLMO, hh:mm																
BWL	20	20:25 [1:01]	15	20:43 [1:27]					0.46	0.76	.55				0.17	
DWL [c]	13	20:33 [1:29]	12	20:40 [1:40]												

NOTE. Raw means and standard deviations are reported. Models were adjusted for marital status

Abbreviations: **BWL** Bright white light; **CES-D** Center for Epidemiological Studies – Depression; **CWS** Cancer Worry Scale; **DLMO** Dim light melatonin onset; **DWL** Dim white light; **FCS** Fatigue Catastrophizing Scale; **IS** Interdaily Stability; **IV** Intradaily Variability; **MFI** Multidimensional Fatigue inventory; **PSQI** Pittsburgh Sleep Quality Index; **RAND-36** RAND 36-item Health Survey; **SES** Self-efficacy Scale; **STAI-6** State Trait Anxiety Index – short form; **VAS fatigue** Visual Analogue Scale fatigue; **WSAS** Work and Social Adjustment Scale.

[a] **T0** baseline; **T1** post intervention; **T2** 3 months after light therapy; **T3** 9 months after light therapy

[b] The effect size was calculated based on the t test statistic (2*t)/(√df); small .20; moderate .50; large .80.

[c] DWL is reference group.

[d] Based on the AIC and BIC criteria, the model with the best fit excluded a random slope and included an autoregressive covariance structure.

[e] identity covariance structure

Supplementary material 6 Pittsburgh Sleep Quality Index subscales analyses

Introduction

The Pittsburgh Sleep Quality Index does not only describe a total score for general sleep quality but also provides scores for seven subscales assessing different aspects of sleep quality: subjective sleep quality, sleep latency, sleep duration, sleep efficiency, sleep disruptions, sleep medication, and daily dysfunctioning. We previously showed that fatigue after cancer was associated with subjective sleep quality and daily dysfunctioning [33]. Therefore, we wanted to further investigate whether these aspects of sleep quality were affected by the light therapy intervention.

Methods

The scores on the subscales of the PSQSI were calculated according to published algorithms. Missing values were replaced by the average score of the completed items of the same scale for each individual provided that at least 50% of the items of a scale had been completed. Subscale scores ranges between 0 (no problems) and 3 (problems). Because of the ordinal character of these outcomes, we used Generalized Estimating Equations to evaluate group differences over time. All models were adjusted for marital status. In case of non-significant group effects, we performed a post-hoc analysis with the exclusion of group to evaluate the time effect.

Results

Figure S6.1 shows the results of the generalized estimating equations per subscale. None of the subscales showed significant differences in change of time between groups indicating that the effect of light therapy was similar in both groups. The post-hoc analyses with the complete samples showed that there was a significant improvement after light therapy on subjective sleep quality, sleep latency, and daily dysfunctioning. This indicates that these aspects of sleep quality improved irrespective of the intensity of light therapy that they used.

Discussion

The results of the subscales of the PSQI showed that BWL showed no superiority to DWL in improving different aspects of sleep quality. When the whole group was evaluated, light therapy did not affect self-reported sleep duration, sleep efficiency, sleep disruptions, and sleep medication use. Light therapy did affect subjective sleep quality, sleep latency, and daily dysfunctioning. This is in line with recent findings that fatigue after cancer was associated with subjective sleep quality and daily dysfunctioning in survivors of (non-)Hodgkin lymphoma[1]. The effect of light therapy on self-reported sleep latency is not in line with the results of the actigraphy assessment (data not shown) in the current study. This indicates that, although the objective time necessary to fall asleep (assessed with actigraphy) did not change,

participants experienced an improvement in the time that they needed to fall asleep (assessed with the self-reported sleep latency scale of the PSQI).

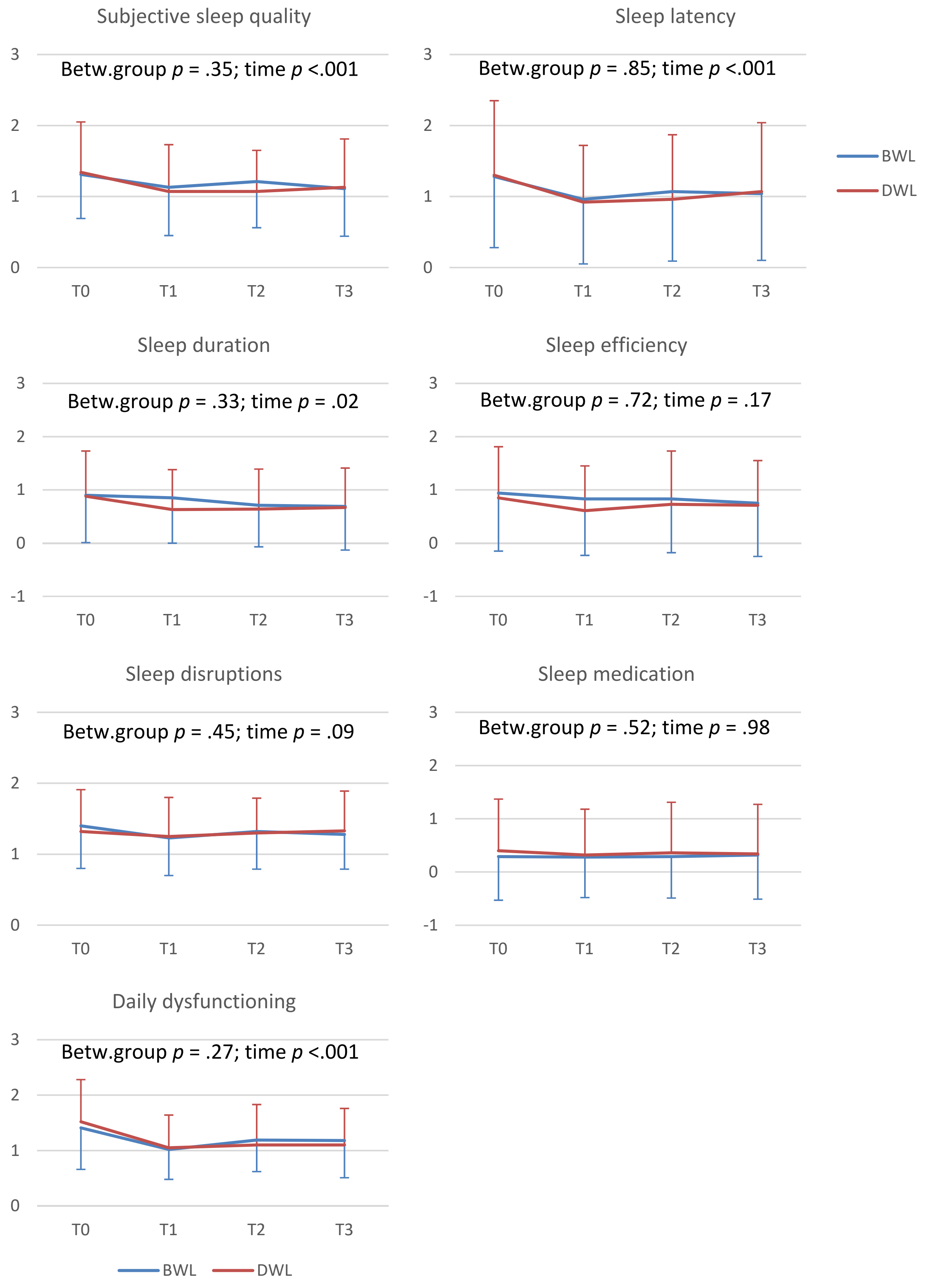

Figure S6.1 Overview of the effect of light therapy on the different subscales of the PSQI.

There were no between group differences on the different aspects of sleep quality (betw.group *p*-value). In the complete sample, we saw a significant improvement on subjective sleep quality, sleep latency, and daily dysfunctioning (time *p*-value).
